# Editorial: The Psychology and Education of Entrepreneurial Development

**DOI:** 10.3389/fpsyg.2020.00027

**Published:** 2020-01-24

**Authors:** Hsiu-Ping Yueh, Yenchun Jim Wu, Wei-Fan Chen

**Affiliations:** ^1^Department of Psychology, Department of Bio-Industry Communication and Development, National Taiwan University, Taipei, Taiwan; ^2^Graduate Institute of Global Business and Strategy, National Taiwan Normal University, Taipei, Taiwan; ^3^Department of Information Sciences and Technology, Penn State Wilkes-Barre, Dallas, TX, United States

**Keywords:** psychology of entrepreneurship, psychological constructs, entrepreneurship in education, entrepreneurial education, entrepreneurial development

Over the past decade, entrepreneurship education has been a significant transformative change happening in Higher Education Institutions worldwide. Entrepreneurship education is different from management education in many ways; students of business school do not necessarily learn entrepreneurship, despite being well-trained with specialized professional competencies, knowledge, and skills related to management and corporate functions. With different core objectives, entrepreneurship education often goes beyond general business education, for it is often connected to innovation education in the disciplines of engineering and technology, and it also is highly related to global, social, political, and technological environments. While many professions have placed great emphasis on developing future entrepreneurs, and young entrepreneurs are being highly encouraged, entrepreneurship education research also needs to focus attention on the psychological nature of entrepreneurial development.

The call for this special Research Topic was intended to elaborate a broad view of the integration in research of psychological factors and educational design in developing entrepreneurial competency as well as in encouraging students to become entrepreneurs. This collection spans a body of work that represents the efforts of 14 original research papers from 44 contributors. As previous psychological studies have examined the psychological constructs used in entrepreneurship, the first paper reveals the influence of a dark triad of personal traits (narcissism, psychopathy, and Machiavellianism) on entrepreneurial intention (Wu W. et al.). Complementarily, several articles examine concepts and models for measuring entrepreneurial intention (Wu W. et al.; Liu X. et al.; Liu F. et al.; Hou et al.; Wang W. et al.; Wang S.-M. et al.). Some articles test the effects of self-efficacy on entrepreneurial intention (Wu W. et al.; Liu X. et al.). To further explore the relationship with more factors, two studies include role models and entrepreneurial passion as factors in the measurement model (Liu F. et al.; Hou et al.) to identify their influences on entrepreneurial intention. In addition, Wei J. et al. adopt an interpretive structure model (ISM model) to identify and analyze entrepreneurial failure learning, and they further propose a multilevel hierarchy of the factors that include both a cognitive dimension–self-efficacy–and an affective dimension–emotion regulation–as two important factors that influence entrepreneurial learning from failure. Wang W. et al. chooses another approach in testing the model of network embeddedness and sense of opportunity identification efficacy on students' social entrepreneurial intention.

Other articles document cognitive factors such as general and specific knowledge, management ability, and business strategy, along with policy issues in entrepreneurial development. For example, Wang looks into law–business compound competency and law–business interdisciplinary entrepreneurship education. Her study suggests that this kind of program should cultivate legal–business intelligence by integrating legal thinking into business logics, building legal awareness in the commercial spirit, preventing legal risk in business competition, and strengthening the application of legal personnel in commercial activities. Another paper, by Zichu, looks at the entrepreneurial opportunity and group intelligence and suggests that joint decision making and constructive controversy are positively related to entrepreneurial opportunity evaluation. For policy issues, Dong et al. explore what entrepreneurial followers need and the difficulties they consider in entrepreneurship cultivation at the start-up stage. Wei X. et al. finds that political skills and entrepreneurial opportunity recognition individually play mediating roles between perceived entrepreneurship education and education.

While 9 studies target university/college students (Wu W. et al.; Liu X. et al.; Liu F. et al.; Hou et al.; Wu W.-H. et al.; Wu and Chen; Wang W. et al.; Wei X. et al.; Wang S.-M. et al.), five papers focus on industry entrepreneurs (Wang; Zichu; Wu W. et al.; Dong et al.; Wei J. et al.). Those studies in the higher education context focus on the curriculum, instruction, and pedagogy in entrepreneurial education. For example, Liu F. et al. adopts storytelling in teaching in entrepreneurship education programs and examines the effects of successful role model stories and failure role model stories together with self-efficacy, entrepreneurial passion, and distance between role model and audience on entrepreneurial intention. Wu W.-H. et al., on the other hand, present the design of an entrepreneurial 9-week social entrepreneurship MOOCs program to teach students with low- to mid- and high-level affective skills and evaluate its effectiveness. Wu and Song implement social media in online entrepreneurial groups of online courses for entrepreneurs, and their research identifies four gratification factors as key incentives for applying social media in such courses. Wu and Chen, employing another approach, present their efforts in partnering with industrial and business experts in entrepreneurial course design and collaborative teaching. They consider this jointly-designed course to be more effective in terms of elevating students' entrepreneurial capabilities and professional competitiveness. Finally, on a more advanced level, Wang S.-M. et al. compare the effects of two paths of entrepreneurial education–a Creativity and Entrepreneurship Program (CEP) and management education–on the development of students' entrepreneurial competencies and intention. They also explore the context limits or facilitations in the entrepreneurship education of university students in different academic disciplines of a management school.

In conclusion, we believe that this Research Topic presents a conceptually broad work and that the papers included in this collection clearly contribute to our understanding of the psychology and education of entrepreneurial development.

Given the fact that higher education institutions worldwide are still increasing the number of entrepreneurial education programs, this Research Topic may not embrace all facets associated with the entrepreneurship research arena. We suggest that future research could focus on the engagement of more psychological and management theories, not only to make better connections between academic training and industrial practice but also to enrich the understanding of the impacts of entrepreneurship on both individual and organizational development. Moreover, it is recommended that more research efforts be directed to those issues needing greater attention, such as the relationships of entrepreneurial development to leadership, path to success, epistemology, and multidisciplinary learning experiences. Furthermore, different research approaches, such as meta-analysis, multi-case study, comparative international entrepreneurship, and mix-method research, are also recommended so as to cast light on entrepreneurship and its impacts on education and society.

## Author Contributions

H-PY conceived of the idea and coordinated the Research Topic. YW and W-FC carried out support tasks for the coordination of the Research Topic and Edition of Articles.

### Conflict of Interest

The authors declare that the research was conducted in the absence of any commercial or financial relationships that could be construed as a potential conflict of interest.

